# Molecular Dynamics Simulation of the Dynamic Mechanical Behavior of FeNiCrMn High-Entropy Alloy

**DOI:** 10.3390/nano15080624

**Published:** 2025-04-19

**Authors:** Haorui Liu, Nana Yang, Shu Xiao, Hu Zhang, Sheng Zhao, Kai Ma, Ning Mi

**Affiliations:** 1 School of Materials Engineering, Longdong University, Qingyang 745000, China; nanayang_material@163.com (N.Y.); nwpu_mn@163.com (N.M.); 2 School of Materials Science and Engineering, Lanzhou University of Technology, Lanzhou 730050, China; mkysys131477@163.com; 3 School of Mechanical & Automotive Engineering, South China University of Technology, Guangzhou 510641, China; zhwysa@163.com (H.Z.); 19818994429@163.com (S.Z.)

**Keywords:** high-entropy alloys, anisotropy, dislocations, stress-strain

## Abstract

High-entropy alloys (HEAs) exhibit excellent properties such as high strength, good ductility, superior corrosion resistance, and thermal stability, making them highly promising for applications in the aerospace, energy, and automotive industries. Among them, the FeNiCrMn HEA demonstrates outstanding corrosion resistance while eliminating the expensive Co element present in the “Cantor” alloy, significantly reducing costs. However, current research on the FeNiCrMn HEA has primarily focused on its corrosion resistance, with relatively limited studies on its mechanical properties. This paper investigated the effects of different crystal orientations, temperatures, and strain rates on the mechanical properties and plastic deformation mechanisms of an equiatomic FeNiCrMn HEA using molecular dynamics simulations. The results revealed that the FeNiCrMn HEA exhibited significant anisotropy under loading along different orientations, with the maximum yield stress observed along the <11-1> direction. During the elastic stage, all crystals maintained a single FCC structure. As strain increased, yielding occurred, accompanied by a sudden drop in stress, which was attributed to the generation of dislocations. The mechanical properties of the FeNiCrMn HEA were highly sensitive to temperature variations. Elevated temperatures intensify atomic thermal vibrations, making it easier for atoms to deviate from their equilibrium positions and facilitating dislocation nucleation and movement. Consequently, the yield strength and yield strain decreased with increasing temperature. In contrast, the yield strength of the FeNiCrMn HEA was relatively insensitive to strain rate variations. Instead, the strain rate primarily affected the alloy’s flow stress. During tensile loading, higher strain rates led to higher dislocation densities. When the stress stabilized, the flow stress increased with the strain rate. These findings provide a theoretical foundation for the future development of FeNiCrMn HEAs.

## 1. Introduction

In 2004, Yeh and Cantor proposed a new alloy design concept, namely high-entropy alloys (HEAs) [1]. Compared with traditional alloys, high-entropy alloys exhibit superior mechanical properties such as high strength, high ductility, high hardness, high wear resistance, and good corrosion resistance, among others [2,3,4,5]. High-entropy alloys, also known as “multi-principal element alloys”, derive their properties from the synergistic effects of multiple elements, rather than reflecting the unique properties of a single element, as in traditional alloys. After solidification, high-entropy alloys do not form numerous intermetallic compounds but typically form solid solution phases with a single structure such as body-centered cubic, face-centered cubic, or hexagonal close-packed structures [6,7,8].

This paper primarily investigated the dynamic mechanical behavior of the FeNiCrMn HEA, which is derived from the classical “Cantor” alloy, specifically the equiatomic CoCrFeMnNi HEA [9]. The practical significance of selecting a FeNiCrMn HEA lies in the realization of a new alloying system that retains the excellent mechanical properties of the original alloy while eliminating the expensive Co element, thereby reducing production costs. Wu et al. [10] reported that when Co is excluded, the mechanical properties of FeNiMnCr18 are similar to or even better than those of the equiatomic CoCrFeMnNi HEA. In the FeNiCrMn HEA, Fe, Ni, and Mn are FCC structure elements, and their inclusion enhances the overall ductility of the alloy. Cr, which is a BCC structure element, provides strength to the alloy. From the perspective of alloy design, this alloy is a HEA with a good balance of strength and ductility. Currently, research on FeNiCrMn HEAs has primarily focused on their corrosion resistance. Elbakh et al. [11] studied the corrosion behavior of the FCC Cr18Mn27Fe27.5Ni27.5 HEA after exposure to molten FLiBe salt at 700 °C for 1000 h, and the results showed that the loss of Mn in the alloy prevented the dissolution of Cr in the molten fluoride salt. Subsequently, Sun et al. [12,13] introduced small amounts of rare earth element lanthanum into CrMnFeNi HEAs. Their studies indicated that the overall corrosion resistance of CrMnFeNiLa0.1 was better than that of CrMnFeNi. However, there is currently a lack of theoretical research on the dynamic loading behavior of this alloy at different temperatures and strain rates.

Molecular dynamics (MD) simulations, as an effective tool for exploring the microstructure and mechanical properties of materials, have been widely used to study the dynamic mechanical properties and deformation mechanisms of materials due to their time- and labor-saving characteristics. Qi et al. [14] analyzed the plastic deformation of single-crystal and polycrystalline CoCrFeMnNi HEAs under tension and compression using MD simulations. The simulation results indicated that during the plastic deformation of the HEA material, phenomena such as FCC to HCP phase transformation, stacking fault, grain refinement, and twinning occurred. Wang et al. [15] simulated the tensile process of crack-free and cracked FeNiCrCoCu HEAs under different temperatures, strain rates, and Cu content. The results showed that increasing temperature and Cu content reduced the mechanical properties of the HEA, while increasing strain rate enhanced the mechanical properties of the HEA. The presence of cracks accelerated the plastic deformation of the HEA. Sun et al. [16] investigated the effect of Ti and Al content and temperature on the mechanical properties of the CoCrFeNiTiAl HEA. The addition of Ti and Al induced the formation of high-temperature strengthening phases in the alloy, significantly improving its mechanical properties, while the material’s strength decreased with increasing tensile temperature. Ting et al. [17] investigated the effect of voids on the mechanical properties of FeNiCrCoCu high-entropy alloys (HEAs) using molecular dynamics simulations. They considered various void sizes, applied strain rates, and temperatures, employing models with one or two voids to examine void evolution. The results indicated that the presence of voids indeed influences the mechanical properties of HEAs. Specifically, the tensile strength decreases with increasing temperature, while it significantly increases with higher strain rates. Similarly, Pan et al. [18] used molecular dynamics (MD) simulations to study the effects of different strain rates and deformation temperatures on the tensile properties of polycrystalline Fe80-xMnxCo10Cr10 (x = 20, 30, 40, 50) medium-entropy alloys (MEAs). Their findings revealed that phase transformation, grain boundary sliding, dislocation slip, and twin formation collectively explain the plastic deformation behavior of these alloys under atomic-scale uniaxial tension. Numerous studies have shown that the plastic deformation behavior of HEAs is primarily attributed to dislocations, stacking faults, twinning, and phase transformation. Research on these mechanisms is crucial for understanding the dynamic mechanical behavior of the FeNiCrMn HEA.

This paper used molecular dynamics simulations to investigate the effects of different crystal orientations, temperatures, and strain rates on the mechanical properties and plastic deformation mechanisms of an equiatomic FeNiCrMn HEA. The aim was to reveal the deformation mechanisms of the FeNiCrMn HEA and provide a theoretical foundation for its future development.

## 2. Computational Details and Model Construction

**Figure 1:**
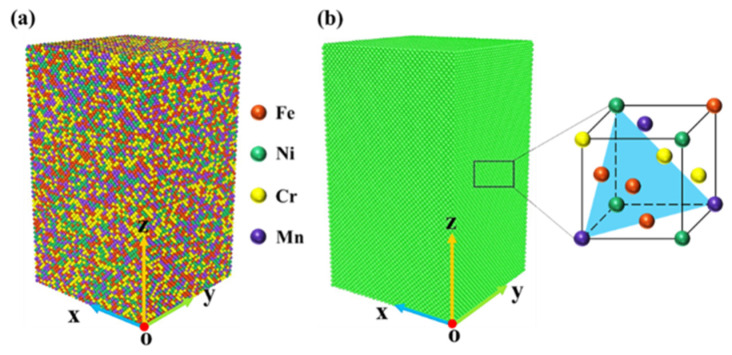
FeNiCrMn high-entropy alloy models. (**a**) Atomic type diagram. (**b**) Crystal structure diagram.

First, the lattice constant of the equiatomic FeNiCrMn HEA was determined using LAMMPS software [19]. Fe was chosen as the base element, and a bulk model of 18 Å × 18 Å × 18 Å was created, with the other elements (Ni, Cr, Mn) randomly substituted into the lattice in proportion, yielding the actual lattice constant. The model size was 108.3 Å × 108.3 Å × 180.5 Å, containing a total of 180,000 atoms, as shown in Figure 1a [20].

The potential function used in this study was the Embedded-atom method (EAM) potential developed by Daw et al. [21], which has been widely used in studies of the plasticity of metallic materials. The EAM potential developed by A. Daramola et al. [22], primarily describing the FCC phase stability, elastic constants, stacking fault energy, dislocations, etc., of the CrFeMnNi HEA, was used. The temperature range for this potential is 0–900 K, which fully met the experimental parameters of this study. During the simulation, periodic boundary conditions were applied in all directions, with a time step of 1 fs. The structure was first optimized using the conjugate gradient (CG) algorithm, and then the NVT ensemble was employed with the Nose–Hoover thermostat to set the initial temperature to 300 K. The relaxation process was carried out in four steps: In the first step, the model was heated from 300 K to 1000 K over a period of 200 ps. In the second step, the model was maintained at 1000 K for 100 ps. In the third step, the model was cooled from 1000 K to 300 K over a period of 200 ps, and in the fourth step, the model was held at 300 K for a total of 100 ps [23]. After these four steps, the model reached equilibrium, and the optimal structure was obtained. The crystal orientations selected were <110>, <1-12>, and <-111>, and the temperature variations were set at 10 K, 300 K, and 600 K. The strain rates were chosen as 10^8^ s^−1^, 10^9^ s^−1^, and 10^10^ s^−1^. Tensile simulations were then performed on the relaxed structures.

The simulation results were analyzed using the visualization software OVITO [24], performing common neighbor analysis (CNA) and dislocation analysis (DXA). DXA was used to identify different dislocation types, and the dislocation density at different strains was calculated using the relevant formulas. CNA was employed to analyze the different phase structures and perform phase composition analysis. As shown in Figure 1b, the initial structure was a single FCC structure.

## 3. Results Analysis

### 3.1. Effect of Crystal Orientation on Mechanical Behavior

**Figure 2:**
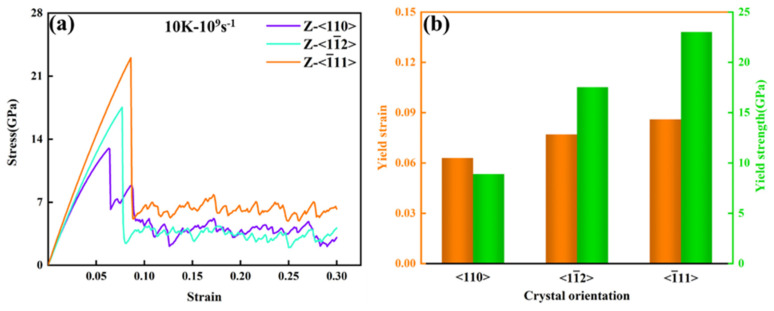
(**a,b**) Tensile stress–strain curves for different crystal orientations and statistical charts of yield strength and yield strain.

The stress–strain curves for uniaxial tensile loading along different crystal orientations of the FeNiCrMn HEA single crystal are shown in Figure 2a. As seen from the figure, there were significant differences among the three crystal orientations during the tensile process, indicating that the FeNiCrMn HEA exhibits pronounced anisotropic characteristics under uniaxial tensile loading.

To facilitate observations, a tensile test was conducted at a temperature of 10 K and a strain rate of 10^9^ s^−1^. By comparing the slopes of the three curves in the elastic stage shown in Figure 2a, it was found that the <-111> direction had the steepest slope, while the <110> direction had the smallest slope. Previous studies have indicated that the slope of the stress–strain curve in the elastic stage represents the elastic modulus. This implies that the elastic modulus is highest in the <-111> direction, followed by <1-12> and <110>. Figure 2b presents the tensile yield strength and yield strain for the three orientations, showing that the <-111> orientation had the highest yield strength. In the elastic stage, the differences in elastic modulus and yield strength among the different crystal orientations were mainly related to the crystal structure of the FeNiCrMn HEA. According to linear elastic theory, the elastic modulus is a physical quantity that characterizes the strength of atomic bonding forces [25]. The closer the atomic packing, the stronger the atomic interactions, making it more difficult to break metallic bonds. Since this HEA had a single FCC structure and the close-packed plane was (111), tensile loading along the <-111> direction resulted in the highest yield strength. As the strain increased, all three curves yielded, and a sudden drop in stress was observed. This was attributed to the generation of crystal defects, which release stress and mark the onset of the plastic deformation stage. With further increases in strain, the tensile curves along the <-111> and <1-12> orientations exhibited stress oscillations after yielding, but the overall stress tended to stabilize. In contrast, the curve for the <110> direction showed an increase in stress after yielding, indicating the occurrence of work hardening.

**Figure 3:**
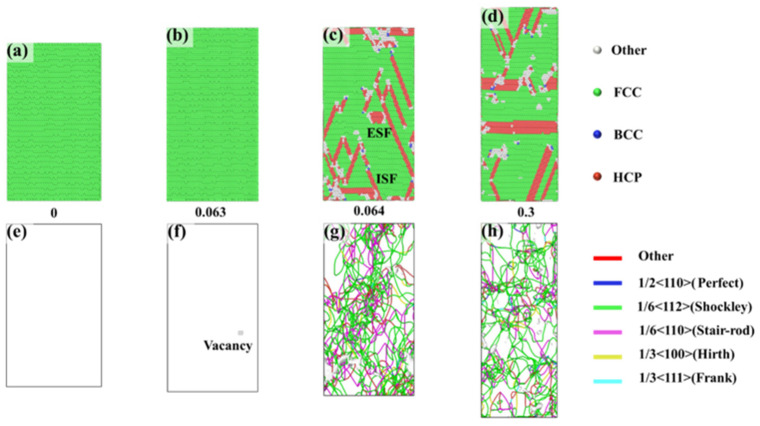
CNA and DXA analyses of the <-111> orientation at different strains: (**a**–**d**) represent the phase structures under different strains, while (**e**–**h**) depict the dislocation distributions under different strains.

To reveal the causes of anisotropy in the FeNiCrMn HEA, the plastic deformation mechanisms during the tensile process were investigated and analyzed in conjunction with the stress–strain curves. Figure 3 presents the CNA and DXA analyses for the <-111> orientation, providing a detailed illustration of the phase structure and dislocation evolution from elastic deformation to plastic deformation. At a strain of 0, the alloy exhibited a single, stable FCC structure (Figure 3a), and no defects were observed within the crystal (Figure 3e), indicating a stable overall structure. As the strain increased to 0.063, the alloy reached its maximum strength. At this point, it still maintained a single FCC structure. However, disordered atoms appeared inside the crystal, leading to the formation of vacancy defects (Figure 3f). With further increases in strain, yielding occurred, and dislocations formed based on the vacancies. We observed that dislocations predominantly nucleated at vacancy sites during the early stages of plastic deformation. This suggests that vacancy aggregation plays an important role in dislocation nucleation. Under applied stress, vacancies tend to diffuse and cluster in regions of high local stress, leading to lattice distortions that act as favorable sites for dislocation formation once the local stress exceeds a critical threshold. While vacancies were a major contributor to dislocation nucleation in our simulations, we acknowledge that other stress-induced mechanisms may have also been involved. The formation of dislocations requires the applied stress to exceed the critical resolved shear stress. When the applied stress exceeds a critical value, numerous dislocations form along the slip planes within the alloy. The primary dislocations observed were Shockley partial dislocations and stair-rod immobile dislocations. These two dislocations influence the entire plastic deformation process. Shockley partial dislocations are mobile dislocations, and their nucleation and motion lead to a reduction in stress. Stair-rod dislocations, on the other hand, are immobile dislocations, and their presence results in an increase in stress. The slip of dislocations results in the formation of intrinsic stacking faults (ISFs) and extrinsic stacking faults (ESFs) [26]. Intrinsic stacking faults (ISFs) and extrinsic stacking faults (ESFs) are two common types of stacking faults in face-centered cubic (FCC) structures, both originating from local disturbances in the atomic stacking sequence. In an ideal FCC crystal, the close-packed layers follow an ABCABC… stacking sequence. If one atomic layer is “removed”, the stacking sequence becomes disrupted—for example, changing to ABCACABC…—this type of fault is referred to as an intrinsic stacking fault (ISF). In contrast, if an extra atomic layer is “inserted” into the normal sequence, resulting in a sequence like ABCBABC…, it is known as an extrinsic stacking fault (ESF). As the strain continues to increase, dislocations proliferate, expand, and annihilate, causing oscillations in stress after yielding, which is consistent with the stress variations shown in Figure 2a. The proliferation of dislocations is accompanied by continuous changes in stacking faults, further influencing the material’s properties.

**Figure 4:**
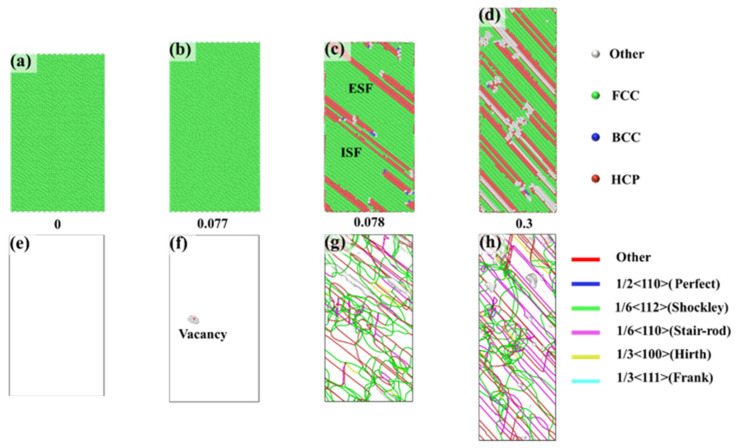
CNA and DXA analyses of the <1-12> orientation at different strains: (**a**–**d**) show the phase structures under different strains, while (**e**–**h**) illustrate the dislocation distributions under different strains.

Figure 4 shows the CNA and DXA analyses for the <1-12> orientation, corresponding to specific points in Figure 2a at different strains. At a strain of 0, the alloy also exhibited a single, stable FCC structure with no defects inside the crystal. As the strain increased to 0.077, disordered atoms (labeled as “Other” atoms) appeared within the crystal, and vacancy defects formed at the center of these disordered atoms (Figure 4f). With further increases in strain, dislocations formed at the vacancies. As these dislocations expanded, a large number of dislocations developed on parallel slip planes. These dislocations were primarily Shockley partial dislocations and stair-rod immobile dislocations. However, the stacking faults induced by the motion of Shockley partial dislocations displayed a parallel distribution, indicating that only one slip system was activated during dislocation slip. The applied strain did not trigger other slip systems. As the strain continued to increase, dislocations proliferated, leading to an overall increase in dislocation density. More stacking faults appeared, and these stacking faults were parallel and remained within the same slip system.

**Figure 5:**
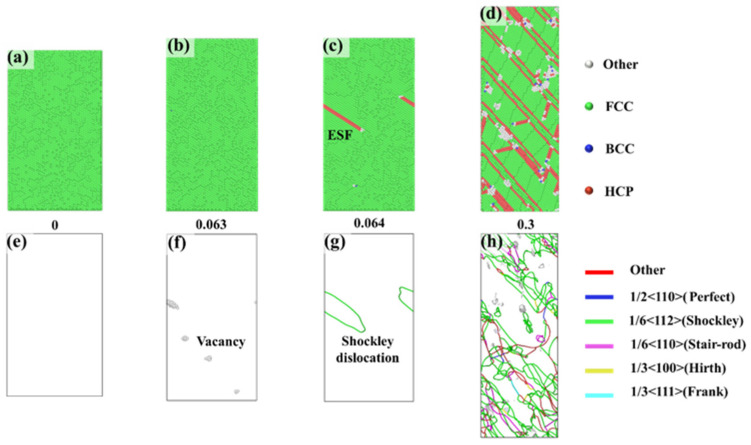
CNA and DXA analyses of the <110> orientation at different strains: (**a**–**d**) represent the phase structures at different strain levels, while (**e**–**h**) depict the dislocation distributions at different strain levels.

The CNA and DXA analyses of the alloy during tensile loading along the <110> orientation are shown in Figure 5. Similarly, at a strain of 0, the entire alloy exhibited a single, stable FCC structure with no defects inside the crystal. When the strain increased to 0.063, multiple regions of disordered atoms appeared within the alloy, forming several vacancies. As the strain increased to the yield strain, dislocations emerged at the vacancy sites, causing a sudden drop in stress. The dislocations that appeared were Shockley partial dislocations, and intrinsic stacking faults (ESFs) were observed at the same locations in the crystal. This clearly indicates that the appearance of ESFs resulted from the movement of Shockley partial dislocations. With further increases in strain, dislocations continued to proliferate but remained confined to the same slip plane, where the generated stacking faults also exhibited a parallel distribution.

**Figure 6:**
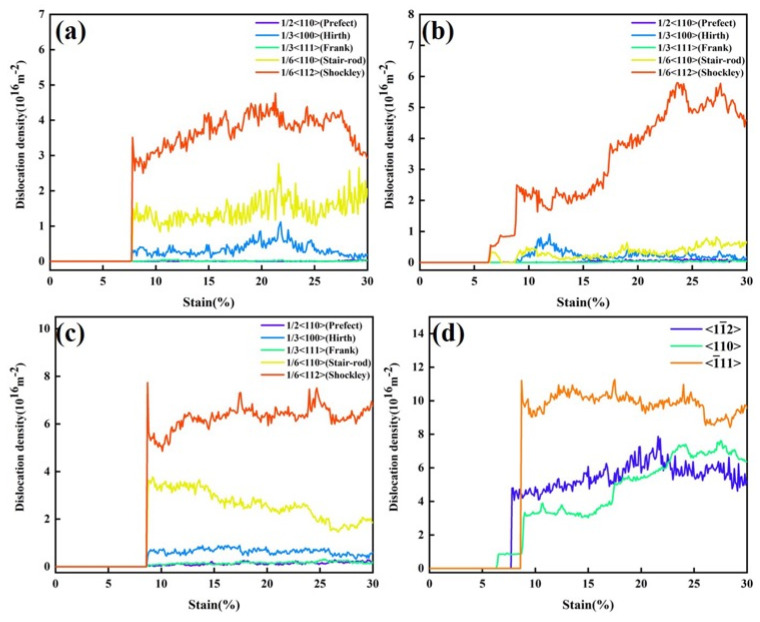
Evolution of dislocation density under different crystal orientations. (**a**) Dislocation evolution for the <$1\bar{1}2$> orientation with different dislocation types. (**b**) Dislocation evolution for the <110> orientation with different dislocation types. (**c**) Dislocation evolution for the <$\bar{1}11$> orientation with different dislocation types. (**d**) Comparison of total dislocation density among the three different crystal orientations.

Figure 6 presents the dislocation density evolution of the FeNiCrMn HEA under tensile loading along three different crystal orientations, and compares the total dislocation density among them. Figure 6a shows the dislocation evolution for the <$1\bar{1}2$> orientation. As strain increases during deformation, plastic flow occurs and dislocations become the primary defects. The dominant dislocation types include 1/6<112>, 1/6<110>, and 1/3<100>. Among them, the 1/6<112> dislocations are Shockley partial dislocations, which can glide along the slip planes, while the other two are sessile dislocations that contribute to strengthening the crystal. Figure 6b illustrates the dislocation evolution for the <110> orientation. Notably, this orientation was dominated by 1/6<112> dislocations, indicating that dislocation slip was more readily activated, which explains the relatively lower strength along this direction. The dislocation evolution for the <$\bar{1}11$> orientation was similar to that of <$1\bar{1}2$>. A closer examination revealed differences in the onset strain for dislocation nucleation among the three orientations. This was attributed to differences in atomic packing density and bonding strength along different directions. In FCC structures, the primary slip system is {111}<110>, with the {111} planes being the closest-packed. Therefore, the <$\bar{1}11$> orientation, which aligns with the close-packed direction, requires a larger strain to initiate plastic deformation, resulting in higher strength [27]. Figure 6d compares the total dislocation density across the three orientations, showing that the <$\bar{1}11$> orientation had the highest dislocation density, which is consistent with dislocation slip theory in FCC crystals.

**Figure 7:**
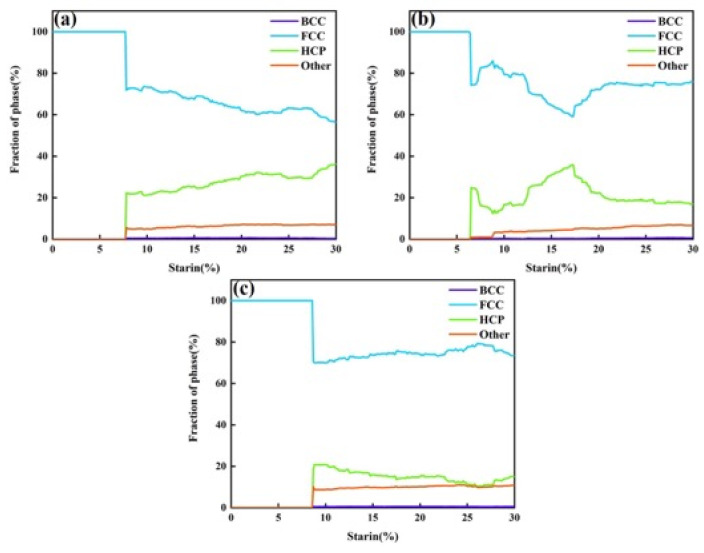
Phase structure evolution under different crystal orientations. (**a**) Phase structure evolution for the <$1\bar{1}2$> orientation. (**b**) Phase structure evolution for the <110> orientation. (**c**) Phase structure evolution for the <$\bar{1}11$> orientation.

To visually understand the phase structure transformation, the atomic structures during the tensile process along the three different orientations were analyzed, as shown in Figure 7. All three structures exhibited a stable FCC configuration in the absence of strain. Comparing this with Figure 6, it can be seen that the strain at which dislocations were generated coincided with the onset of phase transformation, indicating a strong correlation between dislocation motion and phase structure evolution. No BCC atoms were observed in any of the three orientations, indicating that no FCC-to-BCC phase transformation occurred during tensile deformation. The variation in HCP atoms corresponded to the evolution of 1/6<112> dislocations. However, it is worth noting that this trend was not observed in the <110> orientation. As the density of 1/6<112> dislocations increased, the number of HCP atoms decreased. This was mainly due to the generation of 1/6<110> dislocations, which are sessile dislocations formed by the combination of two 1/6<112> partial dislocations. This process is accompanied by the annihilation of ESFs and the increase in ISFs.

### 3.2. Effect of Temperature and Strain Rate on Mechanical Behavior

**Figure 8:**
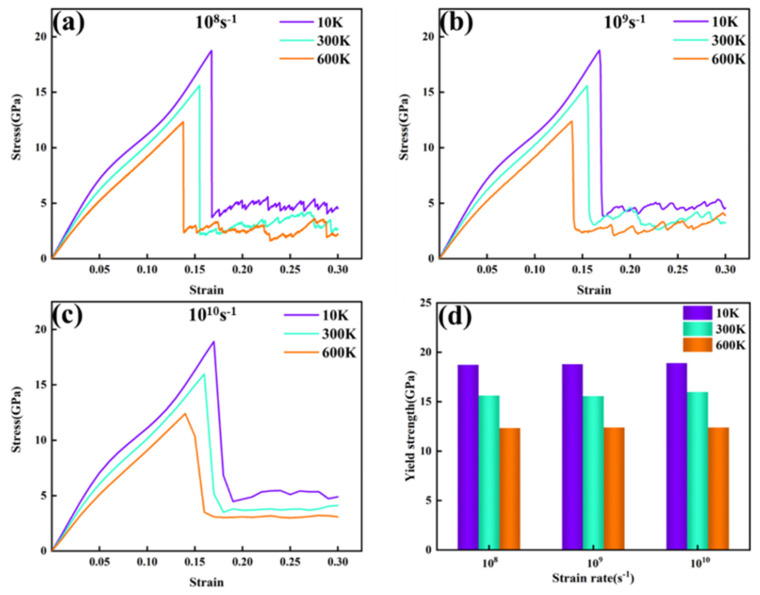
(**a–d**) Stress–strain curves and yield strength charts at different temperatures.

To investigate the effect of temperature on the mechanical properties and deformation behavior of the FeNiCrMn HEA, tensile tests were conducted at a fixed strain rate while varying the tensile temperature. The resulting stress–strain curves for three different temperatures are shown in Figure 8a–c. At a constant strain rate, the yield strength of the alloy exhibited a decreasing trend as the temperature increased [28]. In previous studies, yielding in the stress–strain curve was primarily attributed to the formation and motion of dislocations. As the temperature increases, the strain required for yielding also decreases. This is because higher temperatures lead to stronger thermal vibrations of the atoms, causing larger deviations from their equilibrium positions. These vibrations weaken the atomic bonding forces, thereby reducing the material’s resistance to deformation. Additionally, elevated temperatures lower the critical resolved shear stress required for dislocation slip, enabling more slip paths and facilitating plastic deformation. It is worth noting that after yielding, the stress exhibits a serrated distribution with strain. However, as the strain rate increases, the serrated stress distribution gradually becomes smoother. Each drop in stress corresponds to stress relaxation caused by defect nucleation [29].

**Figure 9:**
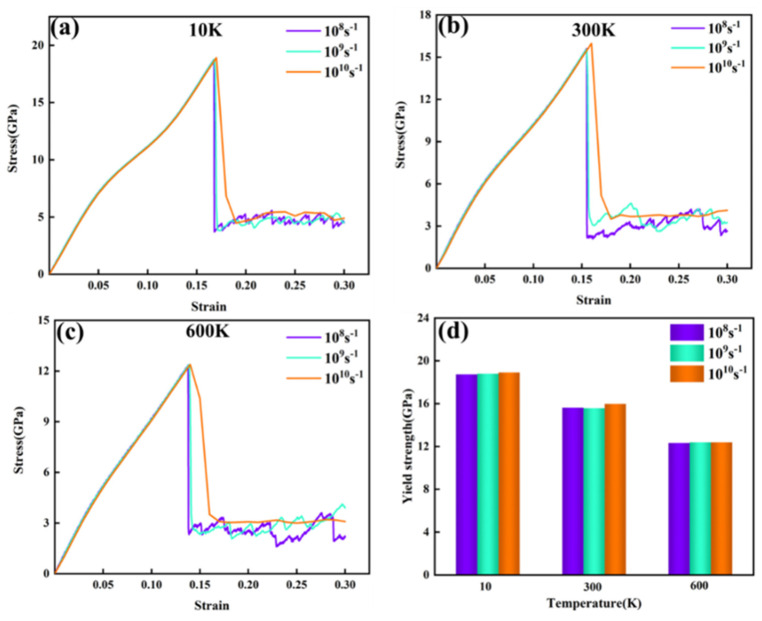
(**a–d**) Stress–strain curves and yield strength charts at different strain rates.

Subsequently, the effect of strain rate on the mechanical properties of the alloy was investigated by keeping the temperature constant and varying the strain rate. The resulting stress–strain curves are shown in Figure 9. Figure 9a–c illustrate the stress–strain curves at different strain rates and temperatures, while Figure 9d summarizes the yield strengths of all curves. From the results, it is evident that at the same temperature, the yield strength was not significantly affected by variations in the strain rate. In other words, this alloy exhibited insensitivity to changes in strain rate. This may be attributed to the fact that the current study was conducted at the nanoscale, and the variations in strain rates examined were relatively small. However, it is noteworthy that as the strain increased, the stress–strain curve at a strain rate of 1010 s^−1^ showed a trend different from those observed at the other two strain rates. After yielding, the stress in the 1010 s^−1^ strain rate curve decreased more gradually and did not exhibit a sharp drop. Furthermore, as the strain increased further, the stress stabilized and did not display a serrated pattern, as shown in Figure 9c,. Previous studies have indicated that the HCP atoms formed during plastic deformation may appear in two ways when a single row of atoms is present: as extrinsic stacking faults (ESFs) or twins (TWIN). When two rows of atoms are observed, they correspond to intrinsic stacking faults (ISFs), and when there are four or more rows, they represent the HCP phase [5].

From the results, it can be deduced that the HCP atoms observed were stacking faults caused by atomic misalignment, consisting of both ISF and ESF types. Atoms identified as “HCP” by common neighbor analysis (CNA) represent local regions with HCP-like coordination. However, most of these atoms appeared in only one or two atomic layers, which, based on prior studies [1], correspond to stacking faults rather than a fully developed HCP phase. Therefore, the HCP atoms in our results were attributed to stacking faults formed during dislocation slip, and not to a true phase transformation. When the strain reached 0.3, multiple stacking faults and cross-slip phenomena were observed within the alloy. At the leading edges of each row of HCP atoms, “other” atoms were predominantly present, indicating that the atomic misalignment originated from the disordered atoms formed during the early stages of tensile deformation. It is also worth noting that with increasing temperature, BCC atoms were observed around the disordered atoms. This phenomenon occurs because higher temperatures reduce the diffusion energy barrier for the FCC-to-BCC transformation [30]. Although the stress–strain curves in Figure 8a–c and Figure 9a–c appeared similar in the elastic region, their post-yield behavior revealed meaningful distinctions. Nevertheless, the overall similarity of the curves highlights the stable elastic modulus and general deformation trends of the FeNiCrMn HEA across the tested temperature and strain rate ranges, indicating that the alloy exhibits robust mechanical stability under varying loading conditions.

**Figure 10:**
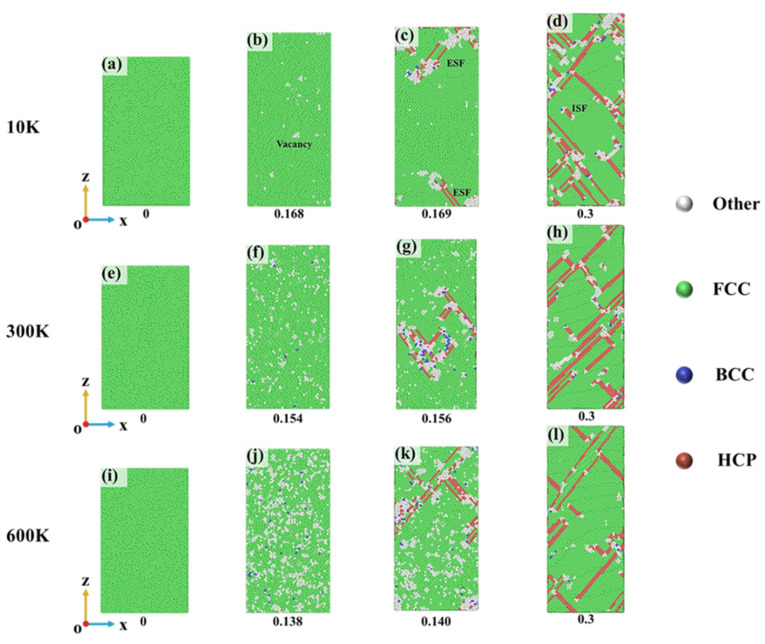
(**a–l**) Phase structure evolution during tensile deformation at different temperatures (CNA analysis).

**Figure 11:**
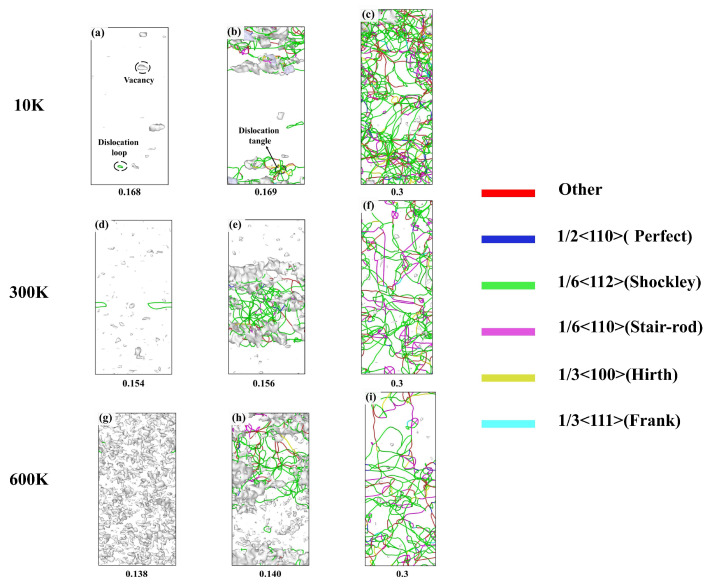
(**a–i**) Dislocation evolution during tensile deformation at different temperatures.

To investigate the uncertainties in the stress–strain curves, a systematic study of the alloy’s microscopic deformation mechanisms was conducted. First, the strain rate of 10^9^ s^−1^ was selected to examine the effect of temperature on the FeNiCrMn HEA. Figure 10 shows the CNA analysis at different strains and temperatures. For clearer observations, snapshots were taken at the Y-interface. From the figure, it can be observed that at a strain of 0, the alloy maintained a single, stable FCC structure at all temperatures. As the strain increased and reached the maximum stress value, disordered atoms appeared, as shown in Figure 10b,f,j. These disordered atoms, labeled as “other” atoms, created localized vacancy defects. With increasing temperature, the proportion of disordered atoms increased. This phenomenon occurred because at the same strain rate, higher temperatures led to faster atomic thermal motion, causing atoms to escape their lattice positions more easily and form disordered structures. As the strain continued to increase, the alloy began to undergo plastic deformation, accompanied by a sudden drop in stress. Meanwhile, HCP atoms emerged in the alloy. The stacking faults were generated by atomic misalignment, which is closely related to the nucleation and motion of dislocations. Investigating the dislocation evolution during the tensile process is a fundamental method for studying the plastic deformation mechanisms of crystals. Therefore, based on the strains observed in Figure 10, Figure 11 provides a statistical analysis of the dislocation variations during the tensile process.

To better observe the dislocations, DXA analysis was performed using the OVITO visualization software [24]. Atoms were removed, leaving only dislocation lines, where lines of different colors represent different dislocation Burgers vectors, as shown in Figure 11. At a strain of 0, the structure was a single, stable FCC phase, which was not further analyzed here. When the strain reached the yield strain, as shown in Figure 11a,d,g, vacancies appeared, and dislocation loops nucleated from these vacancies. The formation of dislocations corresponded to the sudden drop in stress observed in the stress–strain curve in Figure 8b. Since periodic boundary conditions were applied in this study, the dislocation loops in Figure 11d were considered complete loops. As the strain increased, dislocations proliferated, primarily around the initial dislocation. In a comparison with Figure 8, it was observed that the regions of dislocation accumulation coincided with the regions where stacking faults appeared, indicating that the stacking faults were generated by dislocation motion. It is worth noting that not all dislocation motions result in stacking faults. By combining the observations from Figure 10 and Figure 11, it was found that ISFs (intrinsic stacking faults) were caused by the motion of Shockley partial dislocations, while ESFs (extrinsic stacking faults) resulted from two Shockley partial dislocations moving in opposite directions on the same slip plane. As the strain increased to 0.3, dislocation proliferation became more pronounced, predominantly consisting of Shockley dislocations and stair-rod dislocations. Stair-rod dislocations are prone to forming Lomer–Cottrell dislocation locks [31], which have a strengthening effect on the alloy. Therefore, the serrated stress fluctuations observed in the stress–strain curves of Figure 8 after yielding can be attributed to the nucleation and motion of dislocations.

**Figure 12:**
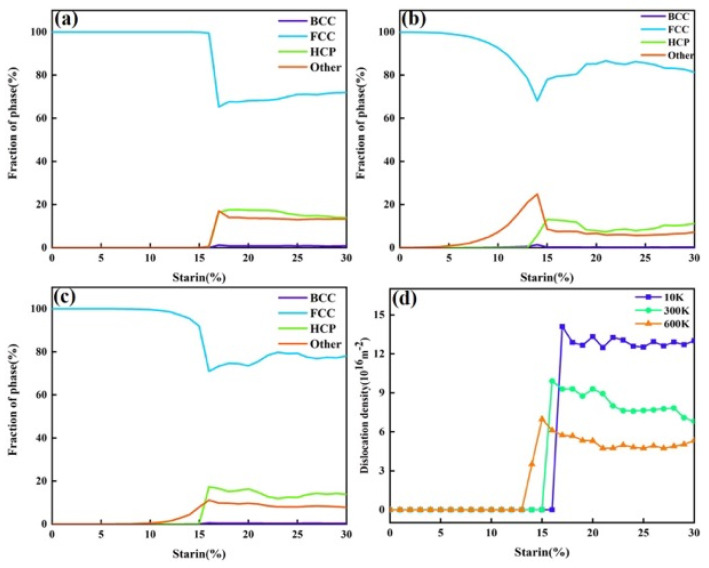
Phase structure and dislocation evolution during tensile deformation at different temperatures. (**a**) Phase structure evolution at 10 K. (**b**) Phase structure evolution at 300 K. (**c**) Phase structure evolution at 600 K. (**d**) Variation in the total dislocation density with strain at the three temperatures.

Figure 12 summarizes the phase structure transformation and dislocation density evolution at three different temperatures. Overall, as the temperature increased, the strain required for the transformation from FCC to HCP atoms decreased, while the proportion of “other” atoms increased. This was mainly due to the intensified atomic thermal vibrations at higher temperatures, which weaken interatomic bonding and make the crystal more susceptible to plastic deformation. As a result, stacking faults induced by dislocation motion are more easily generated, leading to an increased content of HCP atoms—consistent with the observations in Figure 10. The increase in “other” atoms was also attributed to the fact that higher temperatures allow for atoms to escape from their lattice sites more easily, forming vacancies nearby. Atoms surrounding these vacancies are categorized as “other” atoms, and these vacancies are also considered as a major source of dislocation nucleation. Figure 12d shows the dislocation density evolution at different temperatures. As the temperature rose, the dislocation density tended to decrease. This is primarily because the enhanced atomic mobility at higher temperatures allows for dislocations to overcome energy barriers more easily and undergo slip and climb. Dislocations may annihilate through cross-slip or climb out of the slip plane, thereby reducing the overall dislocation density. These results are consistent with the observations in Figure 11.

In summary, the mechanical properties of the FeNiCrMn HEA are highly sensitive to temperature variations. An increase in temperature intensifies the atomic thermal vibrations, making it easier for atoms to deviate from their equilibrium positions. This facilitates the nucleation and motion of dislocations, resulting in a decrease in yield strength and yield strain as the temperature rises. Previous studies have shown that increasing the strain rate can restrict dislocation motion, thereby enhancing the yield strength of materials. However, in this study, the strain rate had a negligible effect on the mechanical properties of the alloy. To investigate this anomalous behavior, CNA and DXA analyses were performed on the alloy model at 300 K under different strain rates.

**Figure 13:**
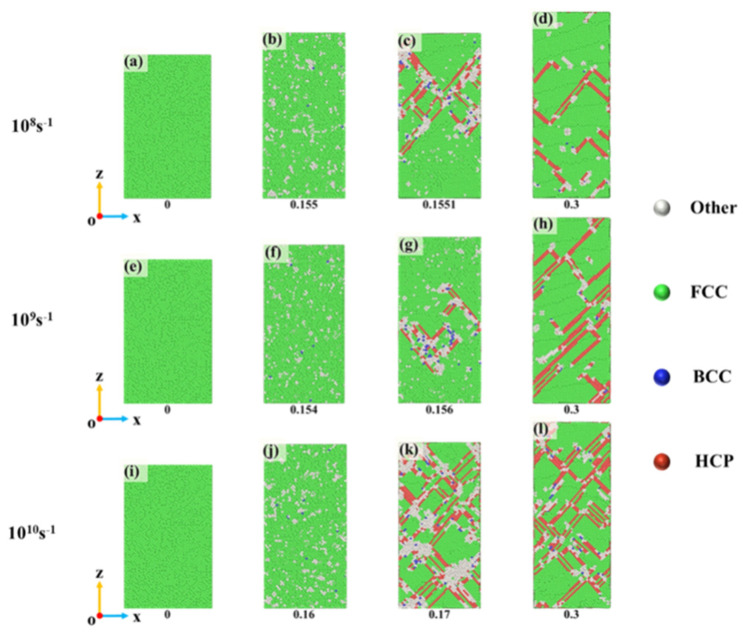
(**a**–**l**) Phase structure evolution during tensile deformation at different strain rates (CNA analysis).

Figure 13 shows the CNA analysis at different strain rates and strains, corresponding to the stress–strain curves in Figure 9. For clarity, snapshots were taken at the Y-interface. Overall, at the same temperature, the variation trends at different strain rates were relatively similar. Initially, disordered atoms appeared, followed by the formation of stacking faults composed of HCP atoms based on these disordered regions. The stacking faults then continued to expand and annihilate. Notably, the locations of stacking fault formation differed at various strain rates. At a strain rate of 10^8^ s^−1^, stacking faults were mainly concentrated in the upper part of the crystal, gradually expanding to the entire crystal as the strain increased. At 10^9^ s^−1^, stacking faults first formed in the middle of the crystal, whereas at 10^10^ s^−1^, stacking faults appeared throughout the entire crystal from the beginning. It was also observed that at a strain of 0.3, higher strain rates led to more stacking faults. Combined with the results in Figure 9, it is evident that at the same temperature, higher strain rates resulted in a higher average flow stress after yielding. This suggests a certain correlation between stacking faults and flow stress. It should be noted that no significant deformation twins were observed in our simulations. This is likely to be due to the high twinning nucleation energy typical of high-entropy alloys (HEAs), which makes twinning less favorable than dislocation slip or stacking fault propagation under the current strain rate and temperature conditions. However, previous experimental studies have reported dense annealing twins in FCC HEAs such as FeNiCoCr and NiCoCr under elevated temperatures. For instance, Wu et al. [27] found that the number of annealing twins increased with the annealing temperature, indicating that thermal activation effectively lowers the energy barrier for twinning. Our group is currently conducting related experiments to explore the twinning behavior during annealing. The preliminary results will be further compared with our simulation findings to distinguish between deformation twins and annealing twins in terms of formation conditions and mechanisms.

**Figure 14:**
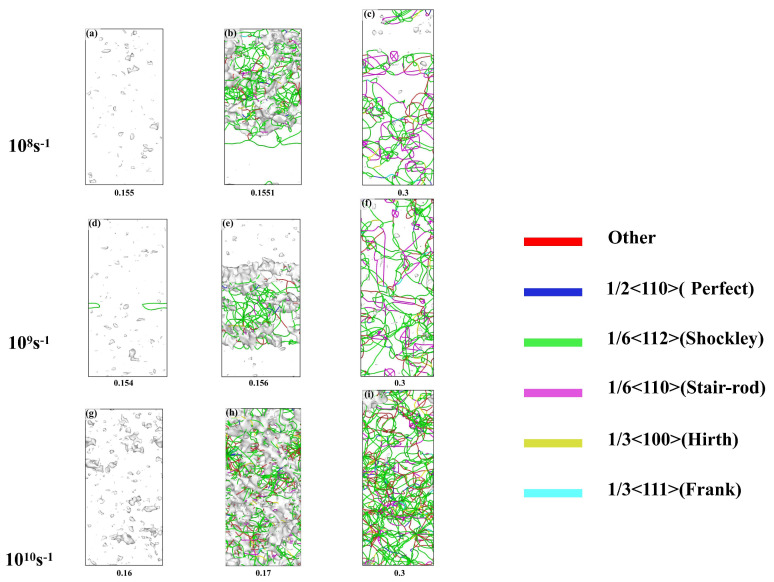
(**a**–**i**) Dislocation evolution during tensile deformation at different strain rates.

Figure 14 illustrates the evolution of dislocations at different strain rates. From the figure, it can be observed that at a strain rate of 10^8^ s^−1^, a large number of vacancy defects were generated at the yield point. Subsequently, the alloy yields and a significant number of dislocations were formed based on the vacancies. The dislocations were primarily 1/6<112> Shockley partial dislocations, which were mainly concentrated in the upper part of the crystal. This corresponded to the locations of stacking faults observed in Figure 13, further confirming that the stacking faults were caused by the motion of Shockley partial dislocations. As the strain increased to 0.3, the dislocations predominantly consisted of 1/6<112> Shockley partial dislocations and 1/6<110> dislocations. Some of the 1/6<110> dislocations were formed by the combination of two 1/6<112> Shockley partial dislocations. The continuous proliferation and expansion of dislocations led to the serrated stress distribution. Among these, the 1/6<110> dislocations were immobile dislocations, and their increasing content contributed to work hardening. Figure 14d–f shows the evolution of dislocations at a strain rate of 10^9^ s^−1^. At the yield point, a large number of vacancies were present, and the first dislocation line was formed. As the strain further increase, dislocations rapidly proliferated and expanded, primarily concentrating in the middle of the crystal. With increasing strain, a large number of dislocations were activated, resulting in a rise in dislocation density. Meanwhile, vacancies nearly disappeared, transforming from point defects into line defects. At a strain rate of 10^10^ s^−1^, a large number of vacancies appeared at the yield point. As strain increased, dislocations were massively activated, spreading throughout the crystal. At a strain of 0.3, dislocations continued to proliferate and annihilate, while stacking faults also underwent constant changes, corresponding to Figure 13i. Overall, the higher the strain rate, the more vacancies are generated at the yield point. As the strain increases, higher strain rates activate more slip systems. At the moment of stress release, a large number of dislocations are generated, resulting in a sudden increase in dislocation density. Higher strain rates correspond to instantaneous dislocation density strengthening, which leads to higher flow stress [32], as shown in Figure 9.

Therefore, the yield strength of this high-entropy alloy is not sensitive to changes in strain rate within this range. Instead, the strain rate affects the alloy’s flow stress. Higher strain rates result in higher dislocation densities, and the increase in dislocation density raises the internal resistance to deformation, thereby increasing the flow stress.

**Figure 15:**
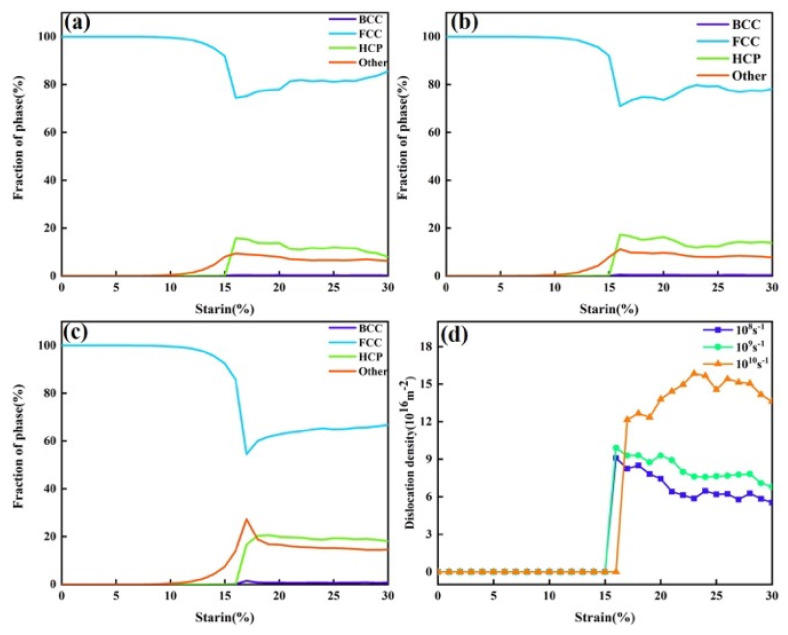
Phase structure and dislocation evolution during tensile deformation at different strain rates. (**a**) Phase structure evolution at a strain rate of 10^8^ s^−1^. (**b**) Phase structure evolution at a strain rate of 10^9^ s^−1^. (**c**) Phase structure evolution at a strain rate of 10^10^ s^−1^. (**d**) Variation in the total dislocation density with strain under the three strain rates.

Figure 15 summarizes the phase structure transformation and dislocation density evolution under three different strain rates. Overall, with increasing strain rate, the proportion of FCC atoms transforming into HCP atoms increased, and the proportion of “other” atoms also rose. As shown in Figure 15d, higher strain rates corresponded to higher dislocation densities. This was mainly because a higher strain rate means that the material undergoes a greater amount of deformation in a shorter time, resulting in increased local internal stress. This provides the driving force for dislocations to overcome energy barriers and activate more slip systems, leading to increased dislocation density. At lower strain rates, dislocations have sufficient time to move and interact—such as annihilating or rearranging through cross-slip or climb—which helps reduce the dislocation density. However, at higher strain rates, dislocation motion and interaction may not keep pace with the deformation process, resulting in more dislocation accumulation and insufficient annihilation, thereby increasing the dislocation density. As previously discussed, the increase in HCP atoms depends on the motion of 1/6<112> dislocations. This trend, along with the dislocation evolution shown in Figure 14, corresponded to the behavior observed in Figure 15d. At the same time, the “other” atoms mainly originated from the disordered atoms surrounding dislocations.

## 4. Conclusions

In this work, molecular dynamics simulations were employed to systematically investigate the effects of crystal orientation, temperature, and strain rate on the mechanical behavior and plastic deformation mechanisms of an equiatomic FeNiCrMn high-entropy alloy (HEA). The main conclusions are summarized as follows:

1. The FeNiCrMn HEA exhibited pronounced anisotropy under uniaxial tensile loading. Among the tested orientations, the <-111> direction showed the highest elastic modulus and yield strength, attributed to the atomic close-packing along the (111) planes in the FCC structure. Dislocation evolution varied with orientation, and deformation was primarily mediated by Shockley partial dislocations and stacking faults. The onset strain for dislocation nucleation also differed among orientations due to variations in atomic bonding and slip resistance.
2. The alloy demonstrated significant sensitivity to temperature. With increasing temperature, atomic thermal vibrations intensified, reducing bonding strength and critical resolved shear stress, which promoted dislocation nucleation and motion. As a result, both the yield strength and yield strain decreased. The dislocation density decreased at higher temperatures due to enhanced dislocation mobility and annihilation. The evolution of phase structure showed increased stacking fault activity and HCP atom formation with temperature, reflecting thermally activated plasticity mechanisms.
3. The yield strength of the alloy was relatively insensitive to strain rate within the simulated range. However, higher strain rates resulted in increased dislocation densities and elevated flow stress after yielding, indicating a typical dislocation strengthening effect. The alloy experienced more severe stress localization and stacking fault accumulation at higher strain rates. These observations suggest that while the strain rate does not significantly alter the onset of yielding, it plays a crucial role in the post-yield hardening behavior.

Overall, the FeNiCrMn HEA exhibited stable elastic behavior and robust plastic deformation responses across a wide range of conditions. These findings provide atomic-level insights into the deformation mechanisms of Co-free HEAs and lay a theoretical foundation for their mechanical design and practical application.

## Data Availability

Data is contained within the article.
